# A Review of Integrative Imputation for Multi-Omics Datasets

**DOI:** 10.3389/fgene.2020.570255

**Published:** 2020-10-15

**Authors:** Meng Song, Jonathan Greenbaum, Joseph Luttrell, Weihua Zhou, Chong Wu, Hui Shen, Ping Gong, Chaoyang Zhang, Hong-Wen Deng

**Affiliations:** ^1^School of Computing Sciences and Computer Engineering, University of Southern Mississippi, Hattiesburg, MS, United States; ^2^Tulane Center of Biomedical Informatics and Genomics, School of Medicine, Tulane University, New Orleans, LA, United States; ^3^College of Computing, Michigan Technological University, Houghton, MI, United States; ^4^Department of Statistics, Florida State University, Tallahassee, FL, United States; ^5^Environmental Laboratory, U.S. Army Engineer Research and Development Center, Vicksburg, MS, United States

**Keywords:** multi-omics imputation, integrative imputation, single-omics imputation, deep learning, autoencoders, machine learning, transfer learning, multi-view matrix factorization

## Abstract

Multi-omics studies, which explore the interactions between multiple types of biological factors, have significant advantages over single-omics analysis for their ability to provide a more holistic view of biological processes, uncover the causal and functional mechanisms for complex diseases, and facilitate new discoveries in precision medicine. However, omics datasets often contain missing values, and in multi-omics study designs it is common for individuals to be represented for some omics layers but not all. Since most statistical analyses cannot be applied directly to the incomplete datasets, imputation is typically performed to infer the missing values. Integrative imputation techniques which make use of the correlations and shared information among multi-omics datasets are expected to outperform approaches that rely on single-omics information alone, resulting in more accurate results for the subsequent downstream analyses. In this review, we provide an overview of the currently available imputation methods for handling missing values in bioinformatics data with an emphasis on multi-omics imputation. In addition, we also provide a perspective on how deep learning methods might be developed for the integrative imputation of multi-omics datasets.

## Introduction

Recent technological developments in high-throughput biology have generated large-scale multi-omics datasets in genomics, epigenomics, transcriptomics, proteomics, metabolomics, metagenomics, and phenomics. Several special collections of publicly available multi-omics datasets have been provided by projects such as Scientific Data (Conesa and Beck, 2019) and The Cancer Genome Atlas (TCGA) (Tomczak et al., 2015). Traditionally, statistical and machine learning (ML)-based approaches have been proposed to identify molecular signatures, discover complicated cellular mechanisms, and predict clinical results from a particular single-omics data source (Mirza et al., 2019). However, single-omics studies often face limitations when attempting to capture the pathological mechanisms of complicated diseases such as cancer, diabetes, and osteoporosis. Overcoming this challenge calls for a systematic approach based on integrative multi-omics analysis that can provide a comprehensive picture of the underlying biological mechanisms.

Multi-omics integration analyses can reveal the connections between different biological factors and provide researchers with a systematic view of the cell and human disease. A recent review presented meaningful integration strategies to elucidate the molecular mechanisms of osteoporosis (Yang T.-L. et al., 2019). By providing a better understanding of the disease pathogenesis, multi-omics integration analyses can prioritize candidates for functional validation experiments with cell lines and/or animal models. Despite the obvious appeal, integration analyses give rise to additional computational challenges beyond those encountered in single-omics studies such as heterogeneous data types, missing data both within and across omics, the curse of dimensionality, imbalanced classes, and issues related to scalability (Mirza et al., 2019).

While many challenges and their solutions have been reviewed elsewhere (Ching et al., 2018; Mirza et al., 2019; Zitnik et al., 2019), to our knowledge, there is no comprehensive and systematic review on missing data in multi-omics integration analyses. The occurrence of missing values is an inevitable problem in multi-omics integrative studies for various reasons, including budget limitations, insufficient sample availability, or experimental constraints. Missing data problems can considerably obstruct downstream analyses in bioinformatics such as clustering of genes and sample classification (Lin et al., 2016). In addition, attempting to handle multi-omics datasets with missing values can hinder integrative analysis as individuals may have missing values within a particular omics dataset or be represented in some omics datasets but not others. Therefore, performing imputation for missing values before the integrative analysis of multi-omics data is an essential and necessary step in discovery and interpretation of the complexities of biology.

In this review, we present a comprehensive and systematic view of the currently available imputation methods for handling missing values in bioinformatics data. This review aims to deliver a summary of current imputation strategies and to serve as a starting point for applying deep learning methods to the imputation of multi-omics datasets. The remaining sections of the paper are organized as follows: (1) an overview of single-omics imputation methods for genotype, gene expression, epigenomic, and proteomic data; (2) an overview of integrative imputation approaches which make use of the inter-omics correlations embedded in the multi-omics datasets; and (3) a discussion that focuses on comparing and contrasting imputation techniques for both single-omics and multi-omics data, followed by an outlook of research trends with an emphasis on multi-model networks and autoencoders.

## Single-Omics Imputation

### Categorizing Single-Omics Imputation Methods

In this section, we briefly introduce single-omics imputation methods and organize them by different data types, including genotype, gene expression, epigenomic, and proteomic data. First, we discuss genotype imputation methods of two broad categories: reference-based and reference-free methods. Second, we review three types of imputation methods for gene expression data: statistical methods, classic ML methods, and deep learning methods. Third, we organize methods for imputing epigenomic data into two categories: statistical methods and deep learning-based methods. Finally, we discuss proteomic data imputation and list examples of methods in three categories: single-digit replacement, local and global similarity-based approaches.

### Genotype Imputation

As an essential tool in genome-wide association studies (GWAS), genotype imputation has facilitated developments in fine-mapping and identification of causal variants, meta-analysis for discovering trait-associated loci, and boosting the statistical power of association tests (Das et al., 2018). Missing values in single-nucleotide polymorphism (SNP) genotyping arrays are very common. They could arise due to a variety of reasons, including deviations from the Hardy-Weinberg equilibrium, low call rates, and the abundance of rare alleles (Chen and Shi, 2019). Several genotype imputation methods are listed in Table 1.

**TABLE 1:** Genotype imputation methods.

	Method	Remarks	Strengths	Limitations
Reference-based	fastPHASE	Haplotype cluster and HMM	Handles samples from multiple subpopulations	Does not estimate recombination rates
	IMPUTE2	MCMC and HMM	First tool to use pre-phasing	Computational complexity
	IMPUTE4	Improvement of IMPUTE2	Faster and more memory efficient	
	BEAGLE 5.0	Graphical model	Handles multi-allelic markers	Computational complexity
	MACH	HMM model		Computational complexity
	FISH	Segmental HMM	No pre-phasing and less computational complexity	
	Minimac3	Improvement of MACH	Engine for web-based imputation servers	
	TUNA, PLINK, UNPHASED, SNPMStat	SNP-tagging approaches	Simpler and faster than HMM-based methods	Only considers local LD structure
Reference-free	SVD, Mean, RF, KNN	Statistical techniques	Easy to implement	Does not model linkage patterns, recombination hotspots, mutations, genotyping errors
	SCDA	Sparse convolutional denoising autoencoder	Deep learning	Hard to interpret the prediction mechanisms

*HMM, Hidden Markov Model; MCMC, Markov-Chain Monte Carlo; FISH, Fast Imputation via Segmental HMM; KNN, K-Nearest Neighbors; SVD, Singular Value Decomposition; RF, Random Forest; SCDA, Sparse Convolutional Denoising Autoencoder.*

Current genotype imputation approaches can be categorized into two groups depending on their requirements for using reference panels (Chen and Shi, 2019). The methods in the reference-free category do not require a reference panel and include common statistical imputation techniques such as replacement with mean, median or mode values, k-nearest neighbors (KNN) (Murti et al., 2019), singular value decomposition (SVD) (Troyanskaya et al., 2001), random forest (RF) (Tang and Ishwaran, 2017), and logistic regression. Recently, with the development of deep learning methods, a sparse convolutional denoising autoencoder (SCDA) approach was proposed to perform genotype imputation without the need of a reference panel (Chen and Shi, 2019).

In contrast, other genotype imputation techniques require a reference panel constructed from whole genome sequencing samples (e.g., 1000 Genomes Project) and have the advantage of making full use of key genetic characteristics such as linkage patterns (or the ordering of genes on chromosomes), mutations, and recombination hotspots (Das et al., 2018). The basic intuition behind these reference-based methods is that short chromosome segments can be shared between any two individuals, as they may be inherited from a distant common ancestor (Das et al., 2018). Das et al. presented a comprehensive overview of genotype imputation from large reference panels (Das et al., 2018). This technique is implemented in the majority of commonly used genotype imputation approaches such as fastPHASE (Scheet and Stephens, 2006), IMPUTE2 (Howie et al., 2009), IMPUTE4 (Bycroft et al., 2017), BEAGLE (Browning and Browning, 2007; Browning et al., 2018), MACH (Li et al., 2010), FISH (Zhang et al., 2014), Minimac3 (Das et al., 2016), PLINK (Purcell et al., 2007), SNPMStat (Lin et al., 2008), TUNA (Nicolae, 2006), and UNPHASED (Dudbridge, 2008). The accuracy of these reference-based imputation methods is mainly determined by the sample size and sequencing coverage of the reference panel, as well as concordance of ethnicity between the individuals in the reference and the GWAS data to be imputed.

Recently, genotype imputation has greatly benefited from the increased availability of publicly available genetic reference panels and is now a standard tool for human genome analysis. In order to make genotype imputation simpler and more accessible, the University of Michigan, Trans-Omics for Precision Medicine (TOPMed) project, and Wellcome Sanger Institute have provided users with three different web-based imputation servers. The Michigan and TOPMed imputation servers are based on Minimac3/Minimac4, while the Sanger imputation server is based on Positional Burrows Wheeler Transform (PBWT) (Durbin, 2014). Although these developments have led to major improvements in imputation accuracy, especially for the most recent TOPMed reference panel which includes > 95,000 deeply sequenced genomes (Kowalski et al., 2019, 000), deep learning-based methods such as SCDA have a lot of utility and may therefore compete with traditional genotype imputation approaches in the future.

### Gene Expression Data Imputation

Transcriptomic profiles are typically acquired using bulk RNA sequencing (RNA-seq) or single-cell RNA sequencing (scRNA-seq), which measure gene expression with different resolutions. The gene expression data imputation methods that we discuss here can be divided into three categories: statistical methods, classic ML methods, and deep learning methods (Table 2).

**TABLE 2:** Gene expression data imputation methods.

	Category	Method	Remarks	Strengths	Limitations
Bulk RNA-seq	Statistical methods	Mean	Row average	Simple	Low accuracy
		KNNimpute	Hot deck imputation	Simple	Difficult to determine K
		GMCimpute	Gaussian mixture clustering with model averaging	Suited to both cross-sectional and time series	Same as KNNimpute
		SEQimpute	MI imputation		Vulnerable to outliers
		GOKNN/GOLLS	Cold deck imputation with gene ontology	Incorporates prior knowledge	
scRNA-seq	Classic ML methods	MAGIC	Neighborhood-based Markov-affinity matrix	Can recover gene-gene relationships	May introduce bias for true zeros
		DrImpute	Clustering based		Ignores gene-level correlation
		scImpute	Gamma-Normal mixture model	Learns gene dropout probabilities	
		SAVER	Bayesian-based model	Quantifies estimation uncertainty	May introduce bias for true zeros
		SAVER-X	Bayesian-based model and autoencoder	Web-based imputation tool	
		VIPER	Weighted penalized regression model	Free of tuning parameters	No uncertainty quantification
		EnImpute	Ensemble learning	Combines eight approaches	
	Deep learning-based methods	SAUCIE	Multi-task deep autoencoder		Difficult to evaluate accuracy
		AutoImpute	Autoencoder-based		
		DCA	Autoencoder with the ZINB loss function		Overfitting
		scVI	Stochastic optimization and VAE	High scalability	
		DeepImpute	Deep neural network-based	Constructs sub-neural networks	

*KNNimpute, K-Nearest Neighbors; GMCimpute, Gaussian Mixture Clustering; SEQimpute, SEQuential imputation; GOKNN, Gene Ontology KNN; GOLLS, Gene Ontology Local Least Squares; MAGIC, Markov Affinity-based Graph Imputation of Cells; SAVER, Single-cell Analysis Via Expression Recovery; VIPER, Variability-Preserving ImPutation for Expression Recovery; SAUCIE, Sparse Autoencoder for Unsupervised Clustering, Imputation and Embedding; DCA, Deep Count Autoencoder; ZINB, Zero-Inflated Negative Binomial; scVI, single-cell Variational Inference; VAE, Variational Autoencoders; DeepImpute, Deep neural network Imputation.*

Traditional bulk RNA-seq (or micro-array) technology analyzes the RNA of an entire cell population, i.e., the gene expression profile that represents the average expression values, weighted by the unknown proportions of different cell types, across the heterogeneous cell population. The popular statistical methods for imputing missing values in bulk RNA-seq datasets can be classified into five general strategies (Gong et al., 2018): (1) impute with the mean; (2) hot deck imputation with methods such as KNNimpute (Troyanskaya et al., 2001); (3) model-based imputation with methods such as GMCimpute (Gaussian Mixture Clustering) (Ouyang et al., 2004); (4) multiple imputation (MI) with methods such as SEQimpute (SEQuential imputation) (Verboven et al., 2007); and (5) cold deck imputation with methods such as GOKNN (Gene Ontology KNN) and GOLLS (Gene Ontology Local Least Squares) (Tuikkala et al., 2006).

Due to the limitations of bulk RNA-seq such as low resolution and inability to study the cellular heterogeneity of a tissue sample, current transcriptome analysis has made the leap from bulk population-based studies to studying gene expression on a single-cell level via scRNA-seq. However, there are particular challenges that arise in scRNA-seq analysis, including high dropout rate and the curse of dimensionality (Zitnik et al., 2019). For scRNA-seq datasets, the observed zeros in the gene expression data matrix are a mixture of true zeros (representing the true gene expression levels in the cells) and dropout zeros (representing the missing data) (Gong et al., 2018). Many classic imputation algorithms have been proposed for handling missing values in scRNA-seq data, including MAGIC (Markov Affinity-based Graph Imputation of Cells) (van Dijk et al., 2018), DrImpute (Gong et al., 2018), scImpute (Li and Li, 2018), SAVER (Single-cell Analysis Via Expression Recovery) (Huang et al., 2018), SAVER-X (Wang et al., 2018), and VIPER (Variability-Preserving ImPutation for Expression Recovery) (Chen and Zhou, 2018). On the other hand, several deep learning-based imputation methods have also been proposed for inferring missing values in scRNA-seq datasets such as SAUCIE (Sparse Autoencoder for Unsupervised Clustering, Imputation and Embedding) (Amodio et al., 2017), AutoImpute (Talwar et al., 2018), DCA (Deep Count Autoencoder) (Eraslan et al., 2019), scVI (single-cell Variational Inference) (Lopez et al., 2018), and DeepImpute (Arisdakessian et al., 2019).

There are a number of unique challenges for gene expression data imputation that are distinct from those for genotype imputation. First, in contrast to genotype imputation, there are seldom external reference panels that can be used to facilitate the imputation of gene expression datasets. Second, unlike genotype data where a given SNP is clearly either genotyped or not genotyped, for gene expression data it is typically impossible to completely distinguish between the true zeros and the dropout zeros. Third, methods for gene expression data imputation have been reported to under-correct or over-correct for data noise in some circumstances, potentially resulting in false positive signals (Andrews and Hemberg, 2019). Therefore, it is well documented that gene expression data imputation is typically far less accurate than genotype imputation (Lähnemann et al., 2020). Furthermore, with the development of different scRNA-seq technologies, the need for gold-standard datasets and methods supporting systematic validation and benchmark analysis is becoming highly pressing (Lähnemann et al., 2020). Recently, Hou et al. presented a timely systematic evaluation for 18 scRNA-seq imputation methods and provided valuable recommendations for improving the downstream analyses (Hou et al., 2020).

While the overwhelming majority of transcriptomic studies evaluate gene expression profiles at a single time point, studying repeated measures of gene expression on the same individuals at multiple time points can provide novel insights into the dynamics of complex biological processes. However, the imputation of time series expression profiles poses unique challenges because the observations from different time points are highly correlated. The autoregressive least squares imputation (ARLSimpute) (Choong et al., 2009) was developed to make use of the correlations between genes as well as the dependencies between time points. The imputation accuracy was shown to be significantly improved compared with traditional imputation techniques which ignore the within sample correlation. Recently, a few other approaches have been proposed for imputation of time series gene expression data such as imputeTS (Moritz and Bartz-Beielstein, 2017), SIMPLEs (Hu et al., 2020), and scIGANs (Xu et al., 2020).

### Epigenomic Data Imputation

Genome-wide maps are constructed by using epigenetic data that describe chromatin accessibility, histone modifications, and DNA methylation (Ernst and Kellis, 2015). Despite the progress toward mapping the epigenome made by large projects such as the Encyclopedia of DNA Elements (ENCODE) and the Roadmap Epigenomics Project, there is still a significant amount of work to be completed in this area (Durham et al., 2018). Due to time, cost and funding constraints, mapping all of the epigenetic markers for every tissue and cell type may not be feasible, indicating the need for accurate imputation approaches.

Overall, current methods for epigenomic data imputation can be categorized into two classes as shown in Table 3: classic statistical methods such as ChromImpute (Ernst and Kellis, 2015), Melissa (MEthyLation Inference for Single cell Analysis) (Kapourani and Sanguinetti, 2019) and PREDICTD (PaRallel Epigenomics Data Imputation with Cloud-based Tensor Decomposition) (Durham et al., 2018), and deep learning-based methods such as Avocado (Schreiber et al., 2018), SCALE (Single-Cell ATAC-seq analysis via Latent feature Extraction) (Xiong et al., 2019) and DeepCpG (Angermueller et al., 2017). However, the existing epigenomic data imputation approaches have several limitations (Ernst and Kellis, 2015). If the occurrence of a marker signal is specific to a limited subset of samples, it will have weak correlation with other markers in the full study sample, leading to poor imputation accuracy at those genomic loci. For instance, imputing transcription factor binding sites is more difficult than predicting epigenetic marks on cell lines since the correlation structure among markers may have a large variability between different samples.

**TABLE 3:** Epigenomic data imputation methods.

	Method	Remarks	Strengths	Limitations
Statistical methods	ChromImpute	Ensemble of regression trees		Does not incorporate genetic variation as an input
	Melissa	Bayesian hierarchical method	Considers local correlations from neighbor CpGs and information across similar cells	No consideration of heterogeneity at the single gene level
	PREDICTD	PARAFAC (Harshman, 1970)/CANDECOMP (Carroll and Chang, 1970) parallelized method with tensor decomposition	3D tensor decomposition and cloud computing	Does not learn non-linear relationships
Deep learning-based methods	Avocado	Tensor factorization and deep neural network	3D tensor decomposition, DNN to learn non-linear relationships	Hyperparameter settings may influence precision and recall
	SCALE	VAE and GMM		
	DeepCpG	Deep learning-based joint model	Uses associations between neighbor CpGs as well as between DNA sequence patterns and methylation states	Does not integrate multi-omics data profiled in the same cell

*Melissa, MEthyLation Inference for Single cell Analysis; PREDICTD, PaRallel Epigenomics Data Imputation with Cloud-based Tensor Decomposition; DNN, Deep Neural Network; SCALE, Single-Cell ATAC-seq analysis via Latent feature Extraction; scATAC-seq, single-cell Assay for Transposase-Accessible Chromatin using sequencing; GMM, Gaussian Mixture Model.*

### Proteomic Data Imputation

In recent years, the field of mass spectrometry (MS)-based proteomics has quickly progressed. Using high-resolution MS techniques, it is possible for modern proteomics studies to detect and quantify vast amounts of proteins and peptides in a single run. These methods can be roughly partitioned into the two broad groups of label-based and label-free quantification (Välikangas et al., 2017). However, label-free methods often face the challenge of a high rate of missing values. For the LC-MS/MS (Liquid Chromatography-Mass Spectrometry)-based approaches, the missing value rate usually varies between 10 and 50%, whereas the rate of peptides (or proteins) having at least one missing value can be extremely high, varying between 70 and 90% (Lazar et al., 2016).

As comprehensively reviewed previously (Webb-Robertson et al., 2015), commonly used statistical imputation approaches for LC-MS proteomics datasets can be grouped into three categories as shown in Table 4: (1) imputation based on single-digit replacement, such as LOD1 (Limit Of Detection), LOD2 and RTI (Random Tail Imputation); (2) imputation based on local structures in datasets, including KNN, LLS (Local Least-Squares), LSA (Least-Squares Adaptive), REM (Regularized Expectation Maximization), and MBI (Model-Based Imputation); and (3) imputation based on global structures, including PPCA (Probabilistic Principal Component Analysis) and BPCA (Bayesian Principal Component Analysis). In general, local similarity-based approaches, such as REM and LSA methods, show the best overall performance in terms of accuracy. However, no single solution dominates all these approaches due to the complicated mechanisms of proteomic data imputation.

**TABLE 4:** Proteomic data imputation methods.

	Method	Remarks	Strengths	Limitations
Single-digit replacement	LOD1	Half of the global minimal intensity among peptides	Simple, good performance for largely left-censored missing values	Poor classification accuracy at peptide and protein levels
	LOD2	Half of the minimal intensity of individual peptide	Same as LOD1	Same as LOD1
	RTI	Random drawing from a truncated normal distribution	Same as LOD1/LOD2	Same as LOD1/LOD2
Local methods	KNN	Weighted average intensity of K most similar peptides	Simple	Difficult to determine K
	LLS	Least-squares based regression model	Automatically estimates K most similar peptides	
	LSA	Weighted LLS		May need to remove features with high missing rate before imputation
	REM	Regularized EM model		May lead to biased estimators and convergence issues
	MBI	ANOVA model		
Global methods	PPCA	PCA and EM		
	BPCA	PCA, Bayesian estimation and EM	Model parameters automatically determined	Assumes global covariance structure which may introduce bias

*LOD1/LOD2, Limit Of Detection; RTI, Random Tail Imputation; LLS, Local Least-Squares; LSA, Least-Squares Adaptive; REM, Regularized Expectation Maximization; MBI, Model-Based Imputation; ANOVA, Analysis of Variance; PPCA, Probabilistic Principal Component Analysis; BPCA, Bayesian Principal Component Analysis; PCA, Principal Component Analysis; EM, Expectation-Maximization.*

## Multi-Omics Imputation

In this section, we first provide a general overview of integrative imputation. Then, we focus specifically on methods that perform imputation using information obtained by combining transcriptomic data with genomic, epigenomic, or proteomic data. Finally, we discuss matrix factor-based imputation and the strengths and limitations of a few methods which use these techniques.

### Integrative Imputation

According to the central dogma of molecular biology, DNA encodes RNA (known as transcription) and RNA encodes proteins (known as translation) (Crick, 1970). This paradigm provides researchers with the most straightforward approach for uncovering the regulatory mechanisms of molecular biology: jointly analyzing both DNA and RNA (or both RNA and proteins) in parallel (Hu et al., 2018).

Similar to single omics data, multi-omics profiles may be collected at either the bulk tissue or single-cell level. Current multi-omics integration approaches for the bulk tissue level data have previously been discussed in detail (Civelek and Lusis, 2014; Huang et al., 2017). From a statistical point of view, integrating multi-omics datasets is equivalent to multi-view learning. Recently, Li et al. presented a comprehensive review focused on the application of various ML methods (including Bayesian models, tree-based methods, kernel methods, network-based fusion methods, ensemble learning, matrix factorization models, and deep neural networks) for the task of integrating multi-view biological data (Li Y. et al., 2018).

On the other hand, the recent maturation of single-cell multi-omics technologies has provided unique opportunities for integrative methods capable of learning from combinations of various data types. These approaches provide researchers with a state-of-the-art tool for profiling different sources of omics data such as DNA, RNA, and proteins on the single-cell level in parallel. Currently, there are a variety of single-cell multi-omics techniques, such as scNMT-seq (single-cell Nucleosome, Methylation and Transcription sequencing) (Clark et al., 2018), CITE-seq (Cellular Indexing of Transcriptomes and Epitopes by sequencing) (Stoeckius et al., 2017), and REAP-seq (RNA Expression And Protein sequencing assay) (Peterson et al., 2017). General strategies for statistical integration of single-cell multi-omics measurements include: (1) multi-view kernel learning, (2) network estimation using the correlation across different cells, (3) multi-view classification with view-specific neural networks, and (4) multi-view matrix factorization (Colomé-Tatché and Theis, 2018).

**FIGURE 1:**
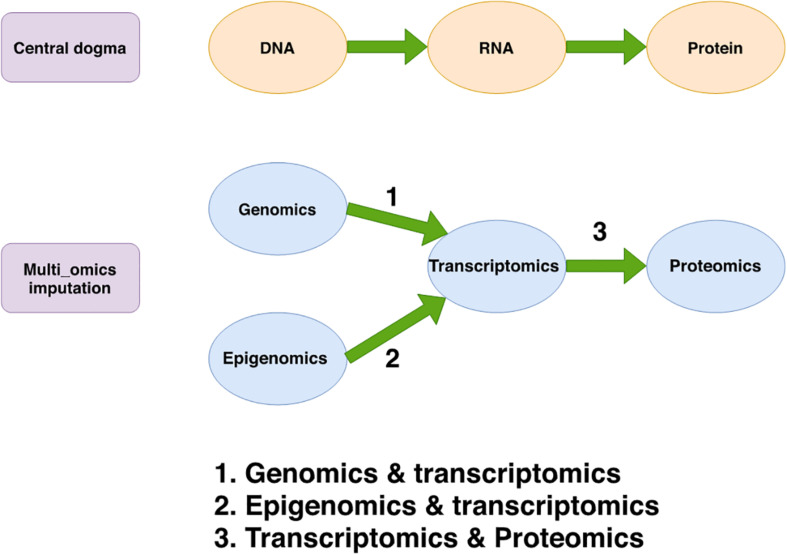
The genetic information flow from DNA to protein via RNA and the interaction between transcriptomics and genomics, epigenomics or proteomics in multi-omics data imputation. The top diagram shows the central dogma of molecular biology in which DNA is transcribed to RNA and then translated to proteins. The bottom diagram shows the integrative relationship between the genomics, epigenomics, transcriptomics and proteomics datasets on which multi-omics data imputation methods are based. These methods are built by combining different omics datasets to perform integrative imputation of missing values. This is shown here by using arrows numbered by the type of data combination they represent. For example, arrow 1 shows that multi-omics imputation can be facilitated by leveraging the correlation between data from both genomics (such as SNP) and transcriptomics (such as gene expression).

However, integrative analysis of multi-omics datasets at both the bulk tissue level and the single-cell level may be hindered by missing values due to technical errors and cost limitations. The underlying principle of multi-omics data imputation is to take advantage of the correlations between different types of biological features measured on the same subjects/cells (as shown in Figure 1). Inspired by the integration strategies for both bulk tissue and single-cell multi-omics, current imputation strategies for missing values in multi-omics datasets involve three distinct approaches: ML-based regression models, transfer learning, and multi-view matrix factorization. Table 5 lists and describes a few implementations of these multi-omics data imputation methods.

**TABLE 5:** Integrative imputation methods for multi-omics datasets.

	Category	Method	Remarks	References
Genomics and transcriptomics	ML-based regression model	PrediXcan	ENet	Gamazon et al., 2015
		S-PrediXcan	GWAS summary statistics	Barbeira et al., 2018
		FUSION	BSLMM	Gusev et al., 2016
		TIGAR	DPR	Nagpal et al., 2018
		CoMM	EM	Yeung et al., 2019
Epigenomics and transcriptomics	ML-based regression model	Lin	Ensemble learning	Lin et al., 2016
		EpiXcan	WENet	Zhang et al., 2019
		TOBMI	KNN	Dong et al., 2019
	Transfer learning	TDimpute	Transfer learning and DNN	Zhou X. et al., 2019
Transcriptomics and proteomics	Transfer learning	cTP-net	SAVER-X and MB-DNN	Zhou Z. et al., 2019
		Seurat v3	Anchor-based transfer-learning	Stuart et al., 2019
Tri-omics	Multi-view matrix factorization	MI-MFA	STATIS	Voillet et al., 2016
		LF-IMVC	Multi-View Clustering	Liu et al., 2019
		MOFA	Unsupervised factorization	Argelaguet et al., 2018

*ML-based regression model, Machine Learning-based regression model; ENet, Elastic-Net model; BSLMM, Bayesian Sparse Linear Mixed Model; TIGAR, Transcriptome-Integrated Genetic Association Resource; DPR, Dirichlet Process Regression; CoMM, Collaborative Mixed Model; WENet, Weighted Elastic Net; TOBMI, Trans-Omics Block Missing data Imputation; cTP-net, single cell Transcriptome to Protein prediction with deep neural network; MB-DNN, Multiple Branch Deep Neural Network; MI-MFA, MI for Multiple Factor Analysis; STATIS, Structuration des Tableaux à Trois Indices de la Statistique; LF-IMVC, Late Fusion Incomplete Multi-View Clustering; MOFA, Multi-Omics Factor Analysis.*

### Integrating Genomic and Transcriptomic Data

Despite the progress of GWAS toward revealing the associations between thousands of genomic loci that harbor genetic variants (typically SNPs) and complex human traits and diseases, the mechanisms governing these associations are still largely undetermined (Wainberg et al., 2019). Recent advancements of transcriptome predictions have led to the rise of transcriptome-wide association studies (TWAS) for the identification of genes with trait-associated expression levels.

TWAS integrate large reference panels including paired genotype and gene expression datasets from the same individuals to uncover gene-trait associations (Wainberg et al., 2019). Usually, there are three steps involved in TWAS. First, a linear regression model is trained to estimate the corresponding weights by using large reference panels, such as GTEx (Genotype-Tissue Expression). Next, these weights are used to predict gene expression from new GWAS datasets. Finally, gene-trait association is performed between imputed gene expression and traits of interest. Current TWAS can be categorized into two classes: individual-level TWAS and summary-level TWAS (Gusev et al., 2016). Individual-level TWAS use effect sizes from reference panels to directly perform expression prediction for genotyped samples. In contrast, summary-level TWAS utilize weighted linear combinations of effect sizes (standardized for SNP traits and considering linkage disequilibrium (LD) effects) to obtain indirect estimates of the relationship between traits and predicted expressions.

Transcriptomic data imputation relies on correlations between genotype and gene expression data and involves the first two steps of TWAS (Nagpal et al., 2018): weights estimation and imputation. In the first step, weights (or cis-eQTL (expression quantitative trait locus) effect sizes) are estimated from the reference panels, which include both genetic and transcriptomic datasets, by considering the following linear regression model:

(1) $$Y_{g}=X\,w+\varepsilon ,\varepsilon ∼N\,\left(0,\delta_{\varepsilon }^{2}\,I\right)$$

where *Y*_*g*_ represents the levels of gene expression (after corrections for some common covariates such as gender, age, and ethnicity), *X* represents the SNP matrix, *w* represents the corresponding weights (or cis-eQTL effect sizes) vector, and ε represents the error term. Next, the genetically regulated gene expression (GReX) is imputed by

(2) $$\hat{G\,R\,e\,X}=x_{n\,e\,w}\,\hat{w}$$

where *x*_*new*_ represents the new GWAS sample dataset. After imputing the GReX, TWAS conduct gene-based tests by testing associations between GReX and the trait of interest. From a gene-based test perspective, TWAS are equivalent to a weighted burden test (Xu et al., 2017; Wainberg et al., 2019), and thus more powerful and adaptive tests such as aSPU (adaptive sum of powered score) (Pan et al., 2014) can be applied to further improve the power.

Several methods have been proposed to improve the estimation of GReX. For example, PrediXcan (Gamazon et al., 2015) is the first and foremost integrative transcriptomic imputation method. The underlying idea is inspired by reference-based genotype imputation methods, which leverage useful information from a large reference panel to impute missing SNPs in the test dataset. PrediXcan is a ML-based method that is trained with large reference panels, including both genotype and gene expression profiles measured on the same individuals, to predict the missing gene expression values based on the genotype data from a new sample. The model uses a set of cis-SNPs that are within 1 Mb upstream and downstream from the transcription region as linear predictors of gene expression. The weights (or cis-eQTL effect sizes) $\hat{w}$ in equation 1 are estimated by using the following Elastic-Net model (ENet) which linearly combines LASSO (*L*_*1*_) and Ridge (*L*_*2*_) penalties to perform variable selection (Zou and Hastie, 2005),

(3) $$\hat{w}=\underset{w}{\text{argmin}}(∥Y_{g}-Xw{∥}_{2}^{2}+\lambda (\frac{1}{2}\left(1-\alpha \right)∥w{∥}_{2}^{2}+\alpha ∥w{∥}_{1}))$$

where ∥⋅∥_*1*_ represents *L*_*1*_ norm, ∥⋅∥_*2*_ represents *L*_*2*_ norm, α*ϵ*[0, 1] represents the weight of the *L*_*1*_ penalty, and λ represents the regulation coefficient for the penalty. After training, these SNP-derived weights are stored in the PredictDB database for the imputation of new datasets, with separate sets of weights for different tissues. The imputed gene expression values may be used for a variety of purposes including gene-trait association studies, as well as other downstream analyses (which significantly depend on the prediction accuracy of GReX) such as the identification of novel therapeutic treatments in GReX-based drug repositioning predictions.

The PrediXcan model is based on the following assumptions: (1) all loci have equal contributions in their roles as possible expression quantitative trait loci (eQTLs), even though they may regulate gene expression through different functional mechanisms, and (2) different alleles have an equal influence on gene expression (Li B. et al., 2018). These assumptions may not necessarily be satisfied in the true biological setting, which may decrease the imputation accuracy. Additionally, PrediXcan only considers cis-SNPs that are located within close proximity to a given gene, and therefore ignores the effects of long-range chromatin interactions and topologically associating domains in the gene expression prediction. Similar to other reference-based imputation approaches, the accuracy of the gene expression imputation is mainly driven by the sample size and ethnicity of the individuals (Fryett et al., 2020). Lastly, prediction models are not available for every tissue and therefore in many cases an alternative biologically related tissue must be chosen. The predicted gene expression in this alternative tissue may not always accurately reflect the expression in the true tissue of interest, which must be taken into consideration when interpreting the proxy tissue results.

In contrast to PrediXcan, which is based on the individual level genotype data, S-PrediXcan (or MetaXcan) (Barbeira et al., 2018) was developed to utilize GWAS summary statistics, which are much more commonly available. In general, three different data sources are needed for this model: a study set, a training set, and a population reference set. Genotype information on the individual level in the study set (or a meta-analysis of several GWAS) is used to calculate the regression coefficients and standard errors between the phenotype and SNPs. The training set is the reference transcriptome dataset where the prediction models for gene expression levels are trained to learn appropriate weights. The training set is also used to calculate the variance and covariance values (LD structure) for the markers utilized when predicting expression levels. However, reference sets covering population-level data (e.g., 1000 Genomes) can be used if training data on the individual level are not available. In the most common cases, only the study set results are needed by the model since both the reference set and training set values can be pre-computed and are available to the user.

FUSION (Gusev et al., 2016) also combines summary association statistics (obtained from large-scale GWAS) with gene expression measurements for the purpose of identifying genes which exhibit expression patterns that are associated with complex traits. The imputed gene expression data can be considered as a weighted linear regression model of genotypes, whose weights are determined by the similarity between SNPs and gene expression datasets (also considering LD among SNPs simultaneously). Specifically, in addition to ENet, FUSION implements several additional ML methods, including LASSO and a Bayesian sparse linear mixed model (BSLMM) (Zhou et al., 2013), to calculate weights from the training datasets. It has been shown that the ENet used by PrediXcan is identical to a Bayesian model that has a Laplace prior and a mixture Gaussian distribution for ***w*** as follows (Zou and Hastie, 2005; Li and Lin, 2010):

(4) $$p\left(w\right)\propto \exp(-\lambda (\frac{1}{2}\left(1-\alpha \right)∥w{∥}_{2}^{2}+\alpha ∥w{∥}_{1}))$$

By contrast, the BSLMM assumes a combination of two normal distributions as the priors for weights estimation (Zhou et al., 2013),

(5) $$w_{i}∼\pi \,N\,\left(0,\left(\delta_{a}^{2}+\delta_{b}^{2}\right)\right)+\left(1-\pi \right)\,N\,\left(0,\delta_{b}^{2}\right)$$

From equation 5 above, BSLMM assumes that all cis-SNPs have a small effect drawn from a normal distribution with a variance of $\delta_{b}^{2}$, and that a subset of cis-SNPs have an extra effect drawn from another normal distribution with a variance of $\delta_{a}^{2}.$ The BSLMM model is equivalent to a Bayesian variable selection regression model (BVSR) or a linear mixed model (LMM) when $\delta_{b}^{2}$ or π is zero, respectively. Therefore, FUSION has great potential for modeling complex genetic architectures by combining the strengths of each model and adaptively selecting the most appropriate one.

TIGAR (Transcriptome-Integrated Genetic Association Resource) (Nagpal et al., 2018) is an improved Bayesian method for imputing gene expression and performing TWAS analysis by utilizing either individual- or summary-level GWAS data. As the former methods such as PrediXcan and FUSION are both based on parametric imputation models, they exhibit limitations when applied to complicated transcriptomic data where the assumptions may be violated. In order to overcome this weakness, TIGAR uses a non-parametric Bayesian model to estimate the prior for cis-eQTL effect sizes with an assumption of a Dirichlet process. This improved Bayesian model (or latent Dirichlet Process Regression (DPR) model), is more general as it involves the parametric priors used by ENet (implemented in PrediXcan) and BSLMM (implemented in FUSION) models respectively, both of which are special cases of this DPR model (Nagpal et al., 2018). The DPR model can robustly extract complicated genetic features of transcriptomic data to significantly improve the imputation accuracy.

To account for uncertainty when imputing the gene expression levels, which has been ignored in the PrediXcan method and may lead to a loss in statistical power, Yeung et al. proposed the collaborative mixed model (CoMM) (Yeung et al., 2019). CoMM addresses the problem of uncertainties in TWAS by jointly modeling the consecutive steps in the imputation model and association tests simultaneously. It fits all parameters in this joint model by using an accelerated expectation-minimization (EM) algorithm (Nelwamondo et al., 2007). The estimated parameters are then used to perform the likelihood ratio test, which evaluates the associations between GReX and the phenotype. However, CoMM depends on GWAS data collected at the individual level. Therefore, CoMM-*S*^2^ (Yang et al., 2019, 2) was developed to make use of GWAS summary statistics (given as estimated SNP effect sizes and their variances) to examine the mechanistic role of genetic variants. Furthermore, CoMM and CoMM-*S*^2^ are both only suitable for single-tissue studies. Recently, two multi-tissue models, UTMOST (Hu et al., 2019) and MultiXcan (Barbeira et al., 2019), have been proposed to combine the gene expression effects across multiple disease related tissues to improve the statistical power of TWAS.

In each of the above methods, the number of genes that can be accurately imputed largely depends on the sample size of the training reference panels and the quality of the training data. For example, PrediXcan only has prediction models available for genes with expression values in GTEx. Therefore, improving the sample sizes of the available references will lead to improved coverage of these imputation methods across the genome. Currently available reference panels include GEUVADIS (Genetic European Variation in Health and Disease), GTEx, DGN (Depression Genes and Networks), METSIM (Metabolic Syndrome in Men), YFS (Young Finns Study), ROSMAP (Religious Orders Study and Memory and Aging Project), NFBC1966 (Northern Finland Birth Cohort 1966) and the 1000 Genomes Project. It is also worth noting that validation of transcriptomic prediction accuracy based on independent datasets is critically important. However, multiple large expression panels are currently not yet available for tissues other than whole blood (Nagpal et al., 2018). Non-trait-related tissues with large expression panels will lead to another challenge of tissue bias for TWAS analysis. In order to address this problem, it is recommended to use an expression panel which has the most trait-related tissues, even if it has a relatively small number of samples compared to others that may be available. On the other hand, using a slightly less related tissue is acceptable if a considerable improvement to the sample size can be obtained. Therefore, the decision to prioritize sample size or to minimize tissue bias should be considered carefully in each study (Wainberg et al., 2019).

### Integrating Epigenomic and Transcriptomic Data

Epigenetic factors such as DNA methylation, histone modification, and chromatin accessibility play an indispensable role in contributing to the discovery of distinct biological functions and complex human diseases. A growing number of results demonstrate that cis-regulatory elements (CREs) such as promoters and enhancers, which exhibit highly enriched levels of risk variants associated with disease, regulate gene expression (Zhang et al., 2019).

By extending single-omics imputation methods to take advantage of useful features from multi-omics data, Lin et al. developed a novel ensemble learning method (Lin) that uses correlations between various types of multi-omics datasets such as miRNA, mRNA, and DNA methylation for multi-omics imputation (Lin et al., 2016). This approach iteratively performs self-imputation (with features within each single-omics modality) and cross-imputation (with features from different omics modalities), followed by a least square regression model to integrate the multiple results from both self- and cross-imputation strategies into a single prediction model.

EpiXcan (Zhang et al., 2019) improves the accuracy of transcriptomic imputation through the incorporation of epigenetic information for the purpose of prioritizing the effect of SNPs on gene expression. In other words, it assigns more weight to SNPs located in CREs, such as promoters and enhancers. There are three steps involved in the implementation of this model: (1) calculate SNP priors by using a hierarchical Bayesian model (qtlBHM) (Li et al., 2016) which jointly leverages REMC (Roadmap Epigenomics Mapping Consortium) (Kundaje et al., 2015) annotation and eQTL summary statistics; (2) transform SNP priors to penalty factors with a mapping function; and (3) predict gene expression by using penalty factors and genotype data in the following weighted elastic net (WENet) equation (Zhang et al., 2019):

(6) $$\hat{w}=\underset{w}{\text{argmin}}(∥Y_{g}-Xw{∥}_{2}^{2}+\lambda \left(\alpha |w{|}_{P}+\left(1-\alpha \right)w^{T}\text{P}w\right))$$

Here, *P* is the weight diagonal matrix whose entries are the penalty factors obtained from rescaled SNP priors. ${\left|\text{w}\right|}_{\text{P}}=\,\sum_{\text{j}=1}^{\text{m}}{\text{P}}_{\text{j}}\,\left|{\text{w}}_{\text{j}}\right|,$ with P_j_ corresponding to the penalty factor of the *j*-th SNP. m represents the number of cis-SNPs. Particularly, if *P*=*I* (equivalent to the identity matrix), the WENet model becomes a standard ENet model. From this point of view, WENet is the more general model and ENet is one of its special cases.

TOBMI (Trans-Omics Block Missing data Imputation) (Dong et al., 2019) is a KNN-weighted approach that allows for imputing trans-omics block missing data. It reliably imputes RNA-seq data by making use of external data from DNA methylation probe datasets. TDimpute (Zhou X. et al., 2019) is a deep neural network (DNN)-based transfer learning approach that imputes missing gene expression data using DNA methylation datasets. It employs a DNN model to recover missing gene expression data by constructing a non-linear mapping between DNA methylation data and gene expression data.

Current integrative imputation methods for epigenomic and transcriptomic data can be broadly categorized into two groups: ML-based regression models (such as Lin (Lin et al., 2016), EpiXcan and TOBMI), and DNN based transfer learning (such as TDimpute). The reference panels of regulatory annotations and gene expression datasets such as REMC, CMC (CommonMind Consortium), GTEx, STARNET (Stockholm-Tartu Atherosclerosis Reverse Network Engineering Task) and TCGA, hold the potential to lead to important insights into epigenomics and disease (Zhang et al., 2019). Since gene-trait associations are mostly detected in strongly relevant tissues, it is recommended to use trait-relevant tissues in order to boost the correlation between GReX of related tissues (Zhang et al., 2019). For the TDimpute model, it can be further improved by integrating prior biological knowledge regarding the gene-gene interaction factors in order to reduce the parameters of the DNN model (Zhou X. et al., 2019).

### Integrating Transcriptomic and Proteomic Data

Recent technological advances such as REAP-seq and CITE-seq protocols allow researchers to simultaneously access transcriptomic expressions and cell surface proteins in the same cell. Cell surface proteins play an increasingly significant role in research areas such as cancer, immunology, and drug development because of their utility as special cellular markers and as potential targets for pharmacological intervention (Bausch-Fluck et al., 2015). However, most current single-cell studies, such as the Human Cell Atlas project, only provide the transcriptome without measurements of the relevant cell surface protein abundances due to technological barriers and cost considerations. Therefore, there is an incentive to explore the possibility of imputing cell surface protein abundances in individual cells by using the cell’s transcriptome (Zhou Z. et al., 2019). In the following, we briefly introduce two representative methods that can be used for integrative imputation of transcriptomic and proteomic data.

cTP-net (single cell Transcriptome to Protein prediction with deep neural network) (Zhou Z. et al., 2019) is a transfer learning-based approach to predict cell surface proteins by using a DNN, which is trained by integrating single-cell multi-omics datasets such as scRNA-seq and given cell surface proteins. It works by performing two main steps: (1) denoise the scRNA-seq matrix by using the SAVER-X model; and (2) impute cell surface protein abundances based on the denoised scRNA-seq data with a mapping from transcriptome to surface protein abundances. This mapping uses a multiple branch deep neural network (MB-DNN) model which can extract multiple gene features reflecting complex cellular environment factors. The input layer for this MB-DNN model is a normalized expression matrix and the output layer is a normalized protein abundance matrix. The first two hidden layers (with dimensions of 1,000 and 128 nodes, respectively) are encoded to learn common shared features across different proteins, such as cell state and cell type. The next hidden layer is dedicated to particular proteins, each of which has 64 nodes. The output layer performs the imputation of surface protein abundances for each of the proteins by reducing the previous hidden layer from 64 nodes to one single node. All layers are fully connected (FC). While the activation function for the output layer is a linear (or an identity) function, all other layers use the rectified linear unit (ReLU) function. The loss function is defined as follows (Zhou Z. et al., 2019):

(7) $$\underset{F}{\text{argmin}}∥Y-F\left(X\right){∥}_{1}$$

where *X* denotes the normalized scRNA count matrix, *Y* denotes the normalized protein abundance matrix, *F* denotes the mapping function, and ∥⋅∥_1_ denotes *L*_1_ norm.

Seurat v3 (Stuart et al., 2019) is an anchor-based transfer learning method for the comprehensive integration of epigenomic, transcriptomic, and proteomic datasets. Through the identification of anchors, which represent pairwise correlations between single cells within different datasets, it can project these datasets into a correlation-shared subspace. These anchors can also help to construct harmonized atlases and map the dataset from a reference into a query. The process of the Seurat v3 model can be briefly summarized using six steps: (1) preprocess data and select features for the reference and query datasets; (2) reduce the dimensionality of both the reference and query datasets into a correlation-shared subspace by performing canonical correlation analysis (CCA), followed by *L*_*2*_ normalization; (3) identify pairs of mutual nearest neighbors (MNN) in the shared space referred to as “anchors” to guide data integration; (4) calculate a score for each pair of anchors accounting for their mutual neighborhood structure for each pair of cells; (5) generate a weight matrix reflecting the strength of association by using the anchor score and the distance between the anchor and the query cell; and (6) transfer information from a reference to the query dataset. One of this method’s applications is to predict cell surface protein abundances based on the cellular transcriptomes in human bone marrow cells by using CITE-seq, which can simultaneously measure immunophenotypes and transcriptomes with single-cell resolution using DNA-barcoded antibodies.

The comparison results of imputation accuracy between Seurat v3 and cTP-net using the benchmark dataset of CITE-seq PBMC (Peripheral Blood Mononuclear Cells) show that the performance of cTP-net is comparable to that of Seurat v3, with cTP-net performing slightly better (Zhou Z. et al., 2019). The cTP-net may have better performance than Seurat v3 in the case of external cell types because cTP-net is trained using a wide variety of cell types, and therefore it may be able to learn features relevant to previously unseen cell types more easily. In contrast, Seurat v3 is based on the nearest neighbors approach which can only be modeled by the training datasets. However, cTP-net has its own limitations (Zhou Z. et al., 2019): (1) it can only be applied to UMI (Unique Molecular Identifier)-based expression input rather than CITE-seq data with TPM (Transcripts Per Million) and RPKM (Reads Per Kilobase Million) expression metrics; and (2) it has a limited ability to generalize to unrelated cell types.

### Matrix Factor-Based Imputation

Several multi-omics imputation methods are based on multi-view matrix factorization techniques. For instance, MI-MFA (Multiple Imputation-Multiple Factor Analysis) (Voillet et al., 2016) involves filling the missing rows in multiple tables using the hot-deck (Joenssen and Bankhofer, 2012) imputation method and then applying MFA to each completed dataset. Finally, it combines these estimated configurations into a compromise configuration using the STATIS method (Structuration des Tableaux à Trois Indices de la Statistique in French). Next, LF-IMVC (Late Fusion Incomplete Multi-View Clustering) (Liu et al., 2019) is designed for multi-omics analysis with each missing dataset seen as an incomplete view. It jointly learns a consensus clustering matrix, imputes each incomplete base matrix, and optimizes the corresponding permutation matrices. Finally, MOFA (Multi-Omics Factor Analysis) (Argelaguet et al., 2018) is an unsupervised statistical approach for integrating multi-omics datasets. It learns low-dimensional common factors for all datasets, which can be used to perform downstream analyses such as data classification, clustering, imputation, and visualization. MOFA takes M datasets as input and decomposes these datasets as (Argelaguet et al., 2018)

(8) $${\text{Y}}^{\text{m}}=Z\,{\text{W}}^{\text{mT}}+\varepsilon^{\text{m}},m=1,\ldots ,M.$$

Where Y^m^ represents each omics data matrix, Z represents the shared factor matrix, W^m^ represents the weight matrix for each omics data m, and ε^m^ represents the residual noise term.

Multi-view matrix factorization methods for multi-omics imputation can be applied to more than two omics datasets. For example, MOFA has been demonstrated to identify factors that elucidate variance throughout multi-omics datasets such as bulk genomic, RNA expression, and DNA methylation data obtained from individuals suffering from chronic lymphocytic leukemia (Argelaguet et al., 2018). However, this model is not free of limitations. First, as a linear model, MOFA lacks the ability to learn the non-linear relationships among features of multi-omics datasets. Although not considered in the current implementation, MOFA could be extended to integrate prior information such as pathway databases in each omics to improve the estimation. Finally, new likelihoods and noise models are needed to expand MOFA to handle datasets with specific statistical properties such as zero-inflated data and binomially distributed data. Recently, the authors presented an improved version of MOFA (MOFA+), which is a more scalable method for the integration of single-cell multi-omics datasets (Argelaguet et al., 2020).

## Discussion

Within the area of single-omics imputation, genotype imputation provides a relatively more mature and standard toolbox for GWAS studies, thanks to the availability of large, public genetic reference panels. In contrast, gene expression data imputation, especially scRNA-seq data imputation, is a more active and challenging field. While scRNA-seq has the advantages of a wide range of technologies for sensitive, combinatorically barcoded or highly multiplexed profiling (Stuart and Satija, 2019), it faces some unique challenges such as the lack of external reference panels and the difficulty in distinguishing between the true zeros and the dropout zeros. As for epigenomic and proteomic data imputation, both are relatively young and fast evolving fields. For integrative multi-omics imputation, gene expression data imputation could serve as an important mediator since the transcriptome is located at the intersection of other omics factors in the central dogma of biology. On the one hand, for strategy 1 (integrating genomic and transcriptomic data) and strategy 2 (integrating epigenomic and transcriptomic data), imputation of transcriptomic data can be facilitated by making use of the correlation between data from both genomics (or epigenomics) and transcriptomics. On the other hand, for strategy 3 (integrating transcriptomic and proteomic data), imputed transcriptomic data can also be used for the purpose of imputing the cell surface protein abundances.

Compared with single-omics imputation, integrative imputation of multi-omics datasets has great potential for helping researchers uncover many informative functional mechanistic pathways, from the original root cause of diseases to functional consequences or relevant interactions. Therefore, it could help to provide a more comprehensive and clearer view for the downstream analysis of multi-omics studies. However, integration of multi-omics data across different samples, experiments, and types of measurements is highly challenging due to the dynamic and complicated mechanisms of biological processes. According to the type of measurements (or omics), samples, experiments (or laboratories), and cells, Lähnemann et al. (2020) proposed five different approaches for the integration of single-cell multi-omics datasets: (1) integrating multiple datasets of single-omics across different samples within the same laboratory; (2) integrating multiple datasets of single-omics information across different samples and laboratories, requiring a stable reference system such as cell atlases; (3) integrating multiple datasets of single-cell multi-omics information; (4) integrating multiple datasets of multi-omics information across different cells at least in the same cell population; and (5) integrating multiple datasets of multi-omics information across different cells and populations, requiring a stable reference system. Additionally, systematically validating and benchmarking different imputation methods for multi-omics datasets presents another challenge. These benchmark methods and datasets should at least meet the following requirements: (1) they need to keep the non-missing values in the original input datasets unchanged after imputation; (2) they need to produce the expected results, such as correctly predicting fake missing values (existing values which were intentionally removed for testing purposes) and minimizing the error ratio; and (3) they need to be robust to system noise and biases.

There are a number of factors that affect the accuracy of multi-omics imputation: missing value mechanism [missing completely at random (MCAR), missing at random (MAR), and missing not at random (MNAR)], missing rate, sample size, degree of overlap of samples, and noise level. Since the principle of multi-omics imputation is to make use of the shared information across the biological datasets, it is critical to use multi-omics datasets measured on the same set of samples. For example, Zhou X. et al. (2019) kept only the subset of samples having both gene expression and DNA methylation data to build the TDimpute model and generate a pan-cancer dataset, which contains 8,856 samples with both gene expression and methylation data for 33 cancers. When there are partially overlapping samples, it may be best to first use only the overlapping samples to train the model (such as TDimpute) and then use this trained model to predict the missing values in new test samples. However, if the number of overlapping samples is small, it will tremendously hinder the training of the model. It is not appropriate to build the multi-omics imputation model with limited or no overlapping multi-omics samples because of the limitation of weak correlation between different omics data not measured on the same individuals. In this case, it is best to revert to single-omics analysis (Lin et al., 2016).

Another emerging challenge is the application of state-of-the-art deep learning methods to multi-omics imputation. Since different omics datasets, such as genomics, epigenomics, transcriptomics, proteomics, and phenomics have their own distinct biological roles and functional mechanisms, it is impossible to use one single, uniform deep learning framework to extract all specific features from these different omics datasets. On the other hand, deep learning methods for multi-omics imputation should have the ability to leverage the correlation or similarity among different omics datasets in order to better impute the missing values in multi-omics datasets. Deep learning methods, especially autoencoders, have already shown great improvements in single-omics imputation. For instance, Chen et al. proposed an autoencoder-based SCDA model for genotype imputation without a reference panel (Chen and Shi, 2019). Another example is AutoImpute, which is also an autoencoder-based imputation approach for inferring missing values in gene expression datasets (Talwar et al., 2018). However, modifying the autoencoder method to handle missing values in multi-omics datasets, which is the subject of our future work, is still a great challenge.

As presented in a recent review of ML methods for integrating multi-view data, the DNN-based multi-model structure is a powerful tool for integrating heterogeneous sets of features with multi-omics data and for capturing their high-level correlations, which can be used for imputation and prediction (Li Y. et al., 2018). Essentially, this involves selecting certain sub-networks to match with a specific type of omics data in order to learn independent features, and then integrating these learned features in a higher layer. These sub-networks make it possible to choose the most suitable deep learning architectures for a specific type of omics data, such as using convolutional autoencoders for genotype imputation and denoising autoencoders for gene expression data imputation. Recently, Sharifi-Noghabi et al. (2019) proposed a DNN-based MOLI (Multi-Omics Late Integration) method to perform drug response prediction by integrating multi-omics datasets such as somatic mutation, copy number variation, and gene expression data. It employs multiple feed forward sub-networks to encode features from each omics dataset, followed by creating a single representation by combining these learned features. Then, it uses a classification network to predict drug response. A highlight of this method is that it is optimized by using a combined cost function including a binary cross-entropy and a triplet loss function.

Multi-model DNN has four attractive advantages for multi-omics data integration (Li Y. et al., 2018). First, the sub-networks can each use different omics data for pretraining before the parameters for the whole network, including the integrative layers and the sub-networks, can be globally fine-tuned. Second, it is possible to simultaneously consider heterogeneous features (depicting differing views) within the integrative layers when performing classification, clustering, imputation, and inference. Third, it is even possible for multi-model networks to learn from samples that have missing values in some omics data. Finally, it is possible to predict profiles for missing omics data using different omics data obtained from the same individual by using generative multi-model networks.

For our future plans, we propose to generate a multi-view autoencoder model for multi-omics imputation by combining multi-model DNN and autoencoders. For example, this novel model may use genotype and gene expression data as two inputs, with at least one of those inputs having missing values, and then produce the two corresponding imputed outputs (without missing values). The two input layers would be separately encoded in the first hidden layer to learn their own specific features before they are concatenated in the second hidden layer. Then, the combined common features could be processed through the bottleneck of the autoencoder in order to leverage the correlation between these two omics data. Finally, the missing values would be imputed after decoding each omics data according to the symmetry structure of the autoencoder. Specifically, we can use different sub-encoder layers, such as convolutional encoders or denoising encoders, and assign different loss functions for different types of omics data in order to better learn their distinct features.

## Author Contributions

H-WD, CZ, and PG conceived and supervised this study. MS and JG carried out literature survey and drafted the manuscript. PG, JL, CZ, and H-WD revised the first draft manuscript. WZ, CW, and HS provided insightful inputs to the original and revised manuscript. All authors contributed to manuscript revisions in response to peer-review comments and approved the final version for publication.

## Conflict of Interest

The authors declare that the research was conducted in the absence of any commercial or financial relationships that could be construed as a potential conflict of interest.
